# Validity of Infant Face Skin Assessment by Parents at Home

**DOI:** 10.31372/20190404.1071

**Published:** 2020

**Authors:** Kaori Yonezawa, Megumi Haruna, Reiji Kojima

**Affiliations:** a Department of Midwifery and Women’s Health, Division of Health Sciences and Nursing, Graduate School of Medicine, The University of Tokyo, Japan; b Department of Health Sciences, School of Medicine, University of Yamanashi, Japan

**Keywords:** infant health, skin assessment, newborn, cross-sectional, skin disease

## Abstract

Parents had better to assess their infant’s skin daily to prevent the development of any skin problems. However, there are no standard methods for assessing infant skin at home. This study aimed to validate the assessment of infant face skin conditions by parents as compared to using skin barrier function clinical tests. In addition, we evaluated the degree of agreement between parents and physicians/midwives when assessing an infant’s skin. A cross-sectional study involving 184 infants aged 3 months was conducted. To evaluate the parents’ infant skin assessment, we used the Neonatal Skin Condition Score (NSCS). On the same day, we evaluated the skin barrier function on the infant’s forehead and cheek, including transepidermal water loss (TEWL), stratum corneum hydration, skin pH, and sebum secretion. Skin barrier function values were correlated with infant skin condition assessed by parents, especially in cases of TEWL of the cheek, for which a moderate positive correlation was found between parental assessment score (ρ = 0.448). In addition, infant with skin problems based on parental assessment had a significantly higher TEWL, lower SCH, and higher skin pH. However, there was weak agreement between parental and physician/midwife assessment. Thus, there was a relationship between parental assessment and skin barrier function; thus, parents can use at-home assessment to assist with infant skin care. In the future, research focused on developing methods of examining infant skin conditions should consider incorporate parental daily skin assessment.

## Introduction

Infants can experience a large variety of skin problems (Matsumoto et al., 2005), which can significantly affect the quality of life for both infants and parents. Methods of assessing infant skin are needed to prevent and reduce the development of dermatological issues. Currently, skin barrier function has been widely regarded as the primary outcome of infant skin research (Blume-Peytavi, Hauser, Stamatas, Pathirana, & Garcia Bartels, 2012). However, within the home setting, symptoms are the most important element of infant skin assessment. Therefore, methods of standardized, at-home infant skin symptom assessment that reflect skin barrier function are needed to improve child care. However, there are two problem to assess infant skin symptoms. First, most infants live at home and cannot be monitored by medical professionals to determine if they have a skin condition. Second, there is a lack of investigations regarding the relationship between skin assessment tools and skin barrier function.

Thus far, almost all research regarding infant skin condition used assessments by physicians, nurses, or researchers who were trained to use any scoring tool (Garcia Bartels et al., 2010; Lavender et al., 2013). Some studies have used the Neonatal Skin Condition Score (NSCS) to evaluate skin condition in terms of erythema, dryness, and breakdown. The NSCS is a validated scale with inter-rater reliability used by medical professionals to assess an infant’s skin condition objectively (Lund & Osborne, 2004). However, it is not feasible for medical professionals to assess infants’ skin condition at home every day. Therefore, if daily assessment of infant skin condition is desirable, parents should be taught to assess this at home.

Parental assessment may be useful to help provide care at home. This is important for not only infant skin research, but also for parents to help assess the necessity of a hospital visit. However, skin conditions are difficult to evaluate correctly. Some parents have anxiety because they overestimate their infant’s skin problem. In contrast, some parents underestimate the skin problem and delay visiting the hospital. A previous study reported a difference in skin dryness when evaluated through objective measurement or maternal assessment (Higuchi, 2017). However, limited studies have investigated parents’ assessment of symptoms of infant skin problems. Therefore, validated assessment methods that can be used by parents are needed.

In addition, no studies were found regarding whether at-home infant skin symptom assessment accurately reflects skin barrier function. Therefore, to validate parental assessment, there is a need for comparison with an objective indicator such as a skin barrier function value. There are some indicators that reflect skin condition, for example, transepidermal water loss (TEWL), stratum corneum hydration (SCH), skin pH, and sebum secretion. However, no studies have considered agreement between parental assessment and skin barrier function.

The aim of this study was to validate parental assessment of infant skin conditions compared to assessment using skin barrier function clinical tests, including TEWL, SCH, skin pH, and sebum secretion. In addition, we evaluated the degree of agreement between parental and physician/midwife assessment to assess inter-rater reliability.

## Methods

### Study Design

This is a cross-sectional study of healthy infants aged 3 months. This study aimed to evaluate the validity of parents’ assessment as part of a randomized control trial (RCT) that evaluated infant skin care (Yonezawa et al., 2018). The study was registered in the University Hospital Medical Information Network Clinical Trials Registry (UMIN000013260). We recruited healthy infants under 4 days of age who were born in a hospital in Tokyo, Japan, at 35 to 42 weeks of gestation between March 2015 and February 2016 for RCT. Inclusion criteria were infants who had no congenital skin disease and who had Asian parents who were literate in Japanese. The research ethics committees of the Graduate School of Medicine, The University of Tokyo, and the hospital where the newborns were recruited from approved the study procedures and protocols (including this study). Written informed consent was obtained from the parents of all the newborns.

### Procedure

The facial skin of infants aged 3 months were assessed. First, parents daily assessed their infants’ skin condition at home and recorded them. A midwife assessed the skin condition and measured the skin barrier function at hospitals when infants were brought for follow-up as part of the skincare RCT. The midwife also took an infants’ face photograph, and a physician later reassessed their skin condition using the photograph.

### Variables

#### Skin Assessments

##### Parental assessment of infant face skin condition score.

To evaluate the parental assessment of the infant face skin condition score based on the NSCS (Lund & Osborne, 2004), parents kept a daily diary for the first 3 months of their infant’s life until the follow-up day. In this study, we used data on the 3-month follow-up day from the skin condition diary that parents kept at home. The NSCS rates a skin condition between 3 and 9 points with no specific cut-off point. In this study, a score of 5 points or higher was considered to represent a clinical skin condition. In this study, the parents used a diary to record their infant’s skin conditions in terms of erythema (none, only 1–2 times, a few times, a moderate number of times, and many times), dryness (none, small amount, moderate amount, and cracking), and breakdown (none and small amount). Since the infants in this study were healthy, few were suspected of having a large degree of skin breakdown, and thus only 1 or 2 points were designated for this category. Therefore, skin conditions were rated on a scale of 3 to 8 points, with a skin problem defined as a score with 5 points or more (Table 1).

**Table 1:** Parental Assessment Score

NSCS (Neonatal Skin Condition Score)		Points assigned for the present study
Erythema	1 = no evidence of erythema 2 = visible erythema <50% body surface 3 = visible erythema >50% body surface	1 point: none, only 1-2 2 points: a few 3 points: moderate, many
Dryness	1 = normal, no sign of dry skin 2 = dry skin, visible scaling 3 = very dry skin, cracking/fissures	1 point: none 2 points: small 3 points: moderate, cracking
Breakdown/excoriation	1 = none evident 2 = small localized areas 3 = extensive	1 point: none 2 points: small (3 points: excluded)

##### Assessment protocol of skin barrier function.

Infant skin barrier function was evaluated at 3 months of age by measuring the values of TEWL (Tewameter TM300, Courage & Khazaka, Cologne, Germany), SCH (Corneometer CM825, Courage & Khazaka), skin pH (Skin-pH-Meter PH905, Courage & Khazaka), and sebum secretion (Sebmeter SM815, Courage & Khazaka). High TEWL and skin pH and low SCH indicate skin barrier dysfunction. These parameters were measured on the infant’s forehead and cheek. The average of two TEWL and sebum secretion measurements and the average of three measurements of the other tests were used. All measurements were conducted in a hospital at room temperature controlled at 24–28 °C at least 5 minutes after the infant entered the room and at least 2 hours after the parents applied any skincare to the infant.

##### Assessment of face skin condition by physicians and midwives.

We used two references to verify validity of parental assessment. First, the physician’s skin condition assessment was conducted by a single pediatric specialist using a photograph, which was obtained by a researcher at the infant’s 3-month check-up day using a digital camera (IXY 620F, Canon, Japan). The pediatric specialist assigned one of the following four categories of skin problems: none (0 point), mild (1 point), moderate (2 points), and severe (3 points). In this study, a skin problem was defined as a score of 1 point or more. Second, the skin condition assessment was conducted by a single midwife who is researcher in person using the NSCS in the same manner as the parental assessment. Therefore, skin conditions were rated on a scale of 3 to 8 points, with a skin problem defined as a score of 5 points or more.

### Statistical Analysis

Correlation between skin condition assessments and skin barrier function were evaluated using Spearman’s correlation coefficient (ρ). We defined ρ value significance level of 0.3–0.5 as a moderate correlation and 0.5–0.8 as a strong correlation. Differences in skin barrier function values between infants with skin problems and those without were evaluated using the Mann–Whitney *U *test. In addition, agreement between parental and physician/midwife skin condition assessment scores were evaluated using the kappa statistic. As we expected a correlation coefficient of over 0.4, we required 102 participants. Therefore, our study achieved adequate sample size.

All statistical analyses were performed using Statistical Package for Social Science, version 24.0 software (IBM Corp., Armonk, NY). All *p*-values were two sided, and a *p*-value < 0.05 was considered statistically significant.

## Results

### Participants

Of the 227 participants recruited from the randomized control trial, 202 infants underwent a 3-month follow-up. Eighteen infants were excluded because their skin conditions were not recorded on the follow-up day or their photo was out of focus. In total, 184 infants were analyzed in this study. The infants’ characteristics are shown in Table 2.

**Table 2:** Infant Characteristics

	All (n = 184)	
Birth season		
Spring	32	17.4%
Summer	51	27.7%
Autumn	55	29.9%
Winter	46	25.0%
Sex: male	101	54.9%
Gestational age (weeks)	39	±1
Birth weight (g)	3002	±355
Family history of AD (*n* = 183)	46	25.1%
Mother had AD (*n* = 183)	28	15.3%
Sibling has AD (*n* = 75)	6	8.0%
Parity: primipara	109	59.2%
Mother’s age (years)	33	±4

Data are presented as *n* (%) or mean ± standard deviation.

AD: atopic dermatitis.

### Comparison of Skin Barrier Function Test and Parental Assessment

First, correlation analysis was conducted between the skin barrier function value and the parental and physician skin condition scores (Table 3). Specifically, the TEWL assessment of the infant’s cheek and the parental assessment had a moderate positive correlation (ρ = .448).

**Table 3:** Correlation Between Assessment of Skin Condition and Skin Barrier Function (n = 184)

		Parental assessment				Midwife assessmenta	Physician assessmentb
		Erythema	Dryness	Breakdown	Total scorea		
TEWL (g/m^2^/h)	Forehead	0.142	0.251	0.168	0.259	0.355	0.154
	Cheek	0.271	0.425	0.260	0.448	0.534	0.179
SCH	Forehead	−0.124	−0.266	−0.202	−0.282	0.456	−0.161
	Cheek	−0.144	−0.236	−0.059	−0.245	0.403	−0.107
Skin pH	Forehead	0.026	0.295	0.059	0.175	0.219	0.154
	Cheek	0.150	0.313	0.096	0.273	0.299	0.127
Sebum	Forehead	0.037	0.063	0.161	0.083	0.032	−0.006
	Cheek	−0.022	0.185	0.181	0.116	0.119	0.028

Spearman’s correlation coefficient. TEWL: transepidermal water loss; SCH: stratum corneum hydration.

^a^Parental assessment and midwife assessment scores were rated between 3 and 8 points.

^b^Point(s) of physician assessment ranged between 0 and 3 points.

Second, a Mann–Whitney *U *test was used to evaluate the difference in skin barrier function values between infants with skin problems and those without skin problems (Table 4). Infants with skin problems had skin barrier dysfunction. Infant skin with skin problems based on parental assessment had a significantly higher TEWL, lower SCH, and higher skin pH.

**Table 4:** Differences in Skin Barrier Function values Between Infants With and Without Skin Problems (n = 184)

		With skin problem^a^ (*n* = 41)	Without skin problem^a^ (*n* = 143)	Effect size (*r*)	*p*-value^b^
TEWL (g/m^2^/h)	Forehead	12.0 (10.6–16.2)	10.7 (8.40–14.6)	0.18	0.016
	Cheek	24.5 (18.0–34.5)	15.5 (10.4–21.8)	0.38	<0.001
SCH	Forehead	54.3 (40.3–62.3)	60.0 (50.3–72.3)	0.23	0.002
	Cheek	48.0 (36.3–60.0)	53.3 (43.0–68.3)	0.18	0.016
Skin pH	Forehead	4.84 (4.64–5.39)	4.76 (4.51–4.96)	0.19	0.009
	Cheek	5.30 (5.05–5.64)	5.12 (4.92–5.35)	0.24	0.001
Sebum	Forehead	20.3 (10.8–38.2)	15.7 (7.3–30.7)	0.12	0.100
	Cheek	3.0 (0.8–8.0)	2.0 (0.7–4.3)	0.11	0.134

Data are presented as a median (interquartile range).

^a^Skin problems were assessed based on parental assessment score (5 or higher).

^b^Mann-Whitney *U* test.

### Agreement between Parental and Physician/Midwife Assessment

Parental and physician assessments had a kappa statistic value of 0.249, which demonstrates a weak level of agreement. One hundred and twelve (60.9%) infants who did not have skin problems were assessed by both the parents and physician, and 20 (10.9%) infants had skin problems.

Next, agreement between parental and midwife assessment had a kappa statistic value of 0.421, which demonstrates a moderate level of agreement. One hundred and fourteen (62.0%) infants who did not have skin problems were assessed by both the parents and midwife, and 28 (15.2%) infants had skin problems.

In addition, agreement between the physician and midwife assessments had a kappa statistic value of 0.424. Furthermore, 14 parents (7.6%) assessed an infant skin problem when neither the physician nor midwife assessed a skin problem and 11 parents (6.0%) assessed no infant skin problem when both the physician and midwife assessed a skin problem. The correlation between parental and physician/midwife assessments is shown in Table 5.

**Table 5:** Correlation of Assessment Between Parents and Physicians/Midwives

	Parental assessment				Midwife scorea
	Erythema	Dry	Breakdown	Total scorea	
Point(s) of physician assessmentb	0.214	0.225	0.089	0.278	0.492
Midwife assessment scorea	0.459	0.433	0.248	0.566	

Spearman’s correlation coefficient.

^a^Parental assessment and midwife assessment scores were rated between 3 and 8 points.

^b^Point(s) of physician assessment ranged between 0 and 3 points.

## Discussion

This study found parents’ assessment to be correlated with objectively measured parameters of skin barrier function in infants. In particular, the novel finding is that if parents assessment showed their infant to have a skin problem, almost all of the skin barrier function values were significantly related to all of the dysfunction variables.

This was especially true for the TEWL of the cheek skin, which exhibited a moderate positive correlation with the parental assessment score. TEWL is an important and standardized indicator of skin barrier function (Ludriksone, Garcia Bartels, Kanti, Blume-Peytavi, & Kottner, 2014); accordingly, the parental assessment tended to agree with an objective value, suggesting the parental assessment is valid. In contrast, a previous study reported a gap between objectively measured skin dryness and maternal assessment (Higuchi, 2017); this suggests that in addition to assessing dryness, assessment of infant skin condition also needs to assess erythema and other variables. Therefore, parental use of an assessment score tool such as the one used in the present study may provide a more valid assessment at home.

In the current study, the correlation between physicians’ assessment using photographs and parental assessment of infants’ skin was weak. Photographic assessment may not be as accurate in the diagnosis of skin disorders in infants as evaluation by physical examination. For example, dynamic aspects of skin disease such as itching and scratching cannot be deduced from a photograph. While many studies have evaluated the contribution of photographs via telemedicine in the field of dermatology, few studies have reported on the difficulty encountered when using photographs to assess infants’ skin (Trettel, Eissing, & Augustin, 2018). Heffner, Lyon, Brousseau, Holland, and Yen (2009) demonstrated the difficulty in photographic diagnosis by showing that while inter-rater reliability was high for dermatologists who used photographs to diagnose pediatric rashes, the correlation was lower when comparison was made with dermatologists who assessed children in person. Our study’s finding of weak correlation between parental and physician assessment of skin breakdown and the moderate positive correlation between parental and midwife in-person assessment highlights the importance of physical examination in the identification of skin disorders among infants. There is a need for a better assessment tool that provides better congruency in assessment between parents and physicians.

This study has some implications. First, parental assessment can be used to monitor an infant’s skin condition continuously for research. Typically, infant skin is usually only assessed by a medical specialist on the specific day of the infant’s check-up appointment—not continuously or every day (e.g., Garcia Bartels et al., 2010; Lavender et al., 2013). We believe there are two potential methods for assessing infant skin daily. First, assessment can be made by the parents. Second, assessment can be made by a specialist via a telescreen or digital photograph. In this study, the parental assessment had a moderate correlation with skin barrier function values than did the photograph assessment made by the specialist. Since the ability to see and touch the infant are important factors in proper skin assessment, daily parental assessment could be a primary outcome of evaluating continuous changes in an infant’s skin condition. In addition, a second implication was the ability for parents to detect possible skin problems for which a medical provider should be consulted. This is useful not only for parents who care infants at home, but also for communication among parents and among medical providers—for example, a newborn-care midwife may provide information to a dermatologist or pediatrician.

This study was limited by the fact that the physician used a photo of the infant’s face to make the assessment rather than using an in-person physical assessment. However, in the future, daily skin assessment could be conducted via telescreens by a specialist. Thus, our study provides valuable information to the development of novel infant skin assessment methods. Second, the NSCS was not designed for use with healthy term infants, because it was developed for use in neonatal intensive care unit. It is possible that other symptoms should be assessed in healthy term infants. This may explain why there was weak agreement between the assessments of parents and physicians/midwives for healthy term infants. In the future, there is a need for a modified assessment tool that has better agreement between parents and physicians/midwives for healthy tern infants.

## Conclusion

Although this study had several limitations, we found that parental assessment of infant face skin has validity to a certain degree in evaluating skin conditions. In the future, research focused on developing methods of examining infant skin conditions should consider incorporating parental daily skin assessment.

## Declaration of Conflicting Interests

The authors declared no potential conflicts of interest with respect to the research, authorship, and/or publication of this article.

## Funding

This study was supported by The Mitsubishi Foundation (grants for Social Welfare Activities on 2013).
